# Supplementary material for: Dynamic transcriptomic profiles of zebrafish gills in response to zinc supplementation
Source: BMC Genomics. 2010 Oct 11;11:553. doi: 10.1186/1471-2164-11-553 (PMC3091702; doi:10.1186/1471-2164-11-553)
Supplement: Additional file 2 — Interactive Direct Interaction Network representing the molecular interactions between zinc, copper, iron, calcium and proteins encoded by transcripts changed by zinc supplementation. Mini web-site containing index.html and hyperlinked pages in subdirectory describing a Direct Interaction Network automatically generated based on curated interactions contained within the proprietary PathwayArchitect database. Ovals represent proteins and the circles symbolize metal ions. Objects are coloured by their abundance in zebrafish at the time-point they were significantly different from the control is a scale from -4 fold (dark green) to +4 fold (dark red). Where significant differences were found at more than one time-point, the colour overlay shows expression at the first instance. Dark blue squares denote 'binding', and light blue squares 'expression'; green squares stand for 'regulation', green diamonds for 'metabolism', and green circles for 'promoter binding'. Arrow heads indicate directionality of the interaction where annotated. All nodes and edges can be further interrogated by selecting the relative area of the image. [file 1471-2164-11-553-S2.zip › PathwayArchitect Zn xs DIN/394823.html]

# BINDING:

|  |  |
| --- | --- |
| Type | BINDING |
| Effect | None |


---

|  |  |
| --- | --- |
| Score | 0 |


---

|  |  |
| --- | --- |
| Reference Count | 30 |


---

|  |  |
| --- | --- |
| Mechanism | Unknown |


---

|  |  |
| --- | --- |
| Reference:0 || Sentence | "In contrast with the results for serum iron, the levels of ferritin, total iron binding capacity (transferrin), zinc, copper and coeruloplasmin were not altered in any subject during the insulin infusion or at 24 h following discontinuation of the infusion." |
| PMID | 3548194 |
| Year | 1987 |
| Species | Human |
| Journal | Acta Endocrinol (Copenh) |
| RefScore | 1 |
| Source | PArchNLP |
  |
|  |  |


---

|  |  |
| --- | --- |
 Reference:1 || Sentence | "Plasma IL-6, TNF alpha, cholinesterase activity (ChE), total iron, unsaturated iron binding capacity (UIBC), and zinc were measured." |
| PMID | 10413184 |
| Year | 1999 |
| Species | Rat |
| Journal | Toxicology |
| RefScore | 1 |
| Source | PArchNLP |
  ||  |  |


---

|  |  |
| --- | --- |
 Reference:2 || Sentence | "The biochemical studies for nutritional evaluation included serum albumin, cholesterol, iron, transferrin, magnesium, zinc, total iron binding capacity (TIBC), and nitrogen balance." |
| PMID | 9486779 |
| Year | 1998 |
| Species | Human |
| Journal | J Surg Oncol |
| RefScore | 1 |
| Source | PArchNLP |
  ||  |  |


---

|  |  |
| --- | --- |
 Reference:3 || Sentence | "Hemoglobin, serum iron, total iron binding capacity, serum and hair zinc concentrations were also determined." |
| PMID | 7234735 |
| Year | 1981 |
| Species | Human |
| Journal | Am J Clin Nutr |
| RefScore | 1 |
| Source | PArchNLP |
  ||  |  |


---

|  |  |
| --- | --- |
 Reference:4 || Sentence | "The mean serum levels of iron, total iron binding capacity, calcium, phosphorus, alkaline phosphatase, zinc, magnesium, vitamin A, and cholesterol were within the normal range in all the three groups." |
| PMID | 6279727 |
| Year | 1981 |
| Species | Human |
| Journal | J Clin Gastroenterol |
| RefScore | 0 |
| Source | PArchNLP |
  ||  |  |


---

|  |  |
| --- | --- |
 Reference:5 || Sentence | "Caution should be exercised when using colorimetric assays for angiotensin-converting enzyme, calcium, iron, magnesium, total iron binding capacity, and zinc in serum samples from patients who have recently received magnetic resonance contrast agents." |
| PMID | 14983944 |
| Year | 2004 |
| Species | Human |
| Journal | Am J Clin Pathol |
| RefScore | 1 |
| Source | PArchNLP |
  ||  |  |


---

|  |  |
| --- | --- |
 Reference:6 || Sentence | "Biochemical parameters included serum albumin, total iron-binding capacity, copper, zinc, and creatinine height index." |
| PMID | 3934976 |
| Year | 1985 |
| Species | Human |
| Journal | Am J Obstet Gynecol |
| RefScore | 0 |
| Source | PArchNLP |
  ||  |  |


---

|  |  |
| --- | --- |
 Reference:7 || Sentence | "MAIN OUTCOME MEASURE: The parameters for determining iron and zinc status were transferrin saturation, zinc protoporphyrin, serum ferritin, hematocrit, serum iron, total iron binding capacity, erythrocyte, and plasma zinc levels (measured by atomic absorption spectrophotometry)." |
| PMID | 11823992 |
| Year | 2002 |
| Species | Human |
| Journal | J Ren Nutr |
| RefScore | 3 |
| Source | PArchNLP |
  ||  |  |


---

|  |  |
| --- | --- |
 Reference:8 || Sentence | "The later decrease in binding of the metals iron and zinc to their transport proteins and the increase in concentrations of the acute phase proteins could be initiated by a common mediator." |
| PMID | 6332122 |
| Year | 1984 |
| Species | Human |
| Journal | J Clin Pathol |
| RefScore | 0 |
| Source | PArchNLP |
  ||  |  |


---

|  |  |
| --- | --- |
 Reference:9 || Sentence | "Liver nonheme Fe was directly affected by dietary Fe and was slightly attenuated by interactions between Cu and Zn, and Zn and Fe." |
| PMID | 10395600 |
| Year | 1999 |
| Species | Rat |
| Journal | J Nutr |
| RefScore | 1 |
| Source | PArchNLP |
  ||  |  |


---

|  |  |
| --- | --- |
 Reference:10 || Sentence | "Mean blood levels of iron, iron binding capacity, calcium, phosphorus, alkaline phosphatase, zinc and magnesium were within normal limits in both groups." |
| PMID | 3005193 |
| Year | 1986 |
| Species | Human |
| Journal | Isr J Med Sci |
| RefScore | 1 |
| Source | PArchNLP |
  ||  |  |


---

|  |  |
| --- | --- |
 Reference:11 || Sentence | "Energy dispersive X-ray fluorescence technique was employed to study the interactions of lead (50 and 100 mg/kg body wt) with K, Fe, Cu, Zn, Br and Rb in rat liver." |
| PMID | 7734922 |
| Year | 1995 |
| Species | Rat |
| Journal | Biometals |
| RefScore | 0 |
| Source | PArchNLP |
  ||  |  |


---

|  |  |
| --- | --- |
 Reference:12 || Sentence | "Manganese, iron, cobalt, nickel, copper and zinc complexes of isatin-beta-thiosemicarbazone (H2L) have been synthesized and spectroscopically characterized The X-ray crystal structures of two nickel complexes, namely [Ni(HL)2]." |
| PMID | 10212992 |
| Year | 1999 |
| Species | Human |
| Journal | J Inorg Biochem |
| RefScore | 0 |
| Source | PArchNLP |
  ||  |  |


---

|  |  |
| --- | --- |
 Reference:13 || Sentence | "The interaction was studied by using analysis of variance (ANOVA), discriminant analysis, and logistic regression to determine the direction of interaction; that is, did dietary Fe affect dietary Zn or did dietary Zn affect dietary Fe? The use of discriminant analysis allowed for using multiple parameters (rather than a single parameter) to determine possible interactions between Fe and Zn." |
| PMID | 12296427 |
| Year | 2002 |
| Species | Rat |
| Journal | Biol Trace Elem Res |
| RefScore | 1 |
| Source | PArchNLP |
  ||  |  |


---

|  |  |
| --- | --- |
 Reference:14 || Sentence | "As determined by ANOVA, only three individual trace elements--liver Fe, Cu, and Mo--were significantly affected by the interaction between Fe and Zn." |
| PMID | 12296427 |
| Year | 2002 |
| Species | Rat |
| Journal | Biol Trace Elem Res |
| RefScore | 1 |
| Source | PArchNLP |
  ||  |  |


---

|  |  |
| --- | --- |
 Reference:15 || Sentence | "For example, an interaction between dietary Fe and Zn affects the weighted linear combination of heart Ca, Cu, K, Mg, Mn, P, and Zn." |
| PMID | 12296427 |
| Year | 2002 |
| Species | Rat |
| Journal | Biol Trace Elem Res |
| RefScore | 2 |
| Source | PArchNLP |
  ||  |  |


---

|  |  |
| --- | --- |
 Reference:16 || Sentence | "RESULTS: At 12 mo, two-factor analysis of variance showed a significant interaction between iron and zinc for weight-for-age z score, knee-heel length, and BSID psychomotor development." |
| PMID | 15321815 |
| Year | 2004 |
| Species | Human |
| Journal | Am J Clin Nutr |
| RefScore | 1 |
| Source | PArchNLP |
  ||  |  |


---

|  |  |
| --- | --- |
 Reference:17 || Sentence | "Duodenal uptake and transfer of 59Fe and 65Zn and absorptive interactions between iron, zinc, cobalt, and copper were studied in sla mice and in genetically normal Swiss albino control mice." |
| PMID | 6498623 |
| Year | 1984 |
| Species | Mouse |
| Journal | Can J Physiol Pharmacol |
| RefScore | 0 |
| Source | PArchNLP |
  ||  |  |


---

|  |  |
| --- | --- |
 Reference:18 || Sentence | "The interaction between zinc and iron does not appear to take place at the site of the genetic defect in sla mice." |
| PMID | 6498623 |
| Year | 1984 |
| Species | Mouse |
| Journal | Can J Physiol Pharmacol |
| RefScore | 0 |
| Source | PArchNLP |
  ||  |  |


---

|  |  |
| --- | --- |
 Reference:19 || Sentence | "Using the InterPro (http://www.ebi.ac.uk/interpro/) database, we categorized 536 redox-sensitive motifs as: 1) active/functional-site cysteines, 2) electron transport, 3) heme, 4) iron binding, 5) zinc binding, 6) metal binding (non-Fe/Zn), and 7) disulfides." |
| PMID | 15138307 |
| Year | 2004 |
| Species | Mouse |
|  | Human |
|  | Rat |
| Journal | Physiol Genomics |
| RefScore | 0 |
| Source | PArchNLP |
  ||  |  |


---

|  |  |
| --- | --- |
 Reference:20 || Sentence | "Further studies are required to define (a) the long-term role of facilitators of iron absorption such as ascorbic acid, (b) the interaction of iron with absorption of divalent trace elements such as zinc, and (c) the effect of iron status on immune function and susceptibility to infection." |
| PMID | 1714697 |
| Year | 1991 |
| Species | Human |
| Journal | Ann Trop Paediatr |
| RefScore | 1 |
| Source | PArchNLP |
  ||  |  |


---

|  |  |
| --- | --- |
 Reference:21 || Sentence | "CBC, serum albumin, serum total protein, serum iron, iron binding capacity, serum zinc and copper, and serum vitamin C were studied weekly for 4 weeks and biweekly for 8 weeks." |
| PMID | 1634717 |
| Year | 1992 |
| Species | Human |
| Journal | J Am Geriatr Soc |
| RefScore | 0 |
| Source | PArchNLP |
  ||  |  |


---

|  |  |
| --- | --- |
 Reference:22 || Sentence | "Blood samples were analysed for haemoglobin, serum iron, total iron binding capacity (TIBC), ferritin, retinol and zinc." |
| PMID | 10099942 |
| Year | 1999 |
| Species | Human |
| Journal | Eur J Clin Nutr |
| RefScore | 0 |
| Source | PArchNLP |
  ||  |  |


---

|  |  |
| --- | --- |
 Reference:23 || Sentence | "RESULTS: At the end of the study, at the age of 13 weeks, weight and length gain, and most serum parameters (iron, iron binding capacity, calcium, phosphorus, protein, prealbumin, zinc) were comparable in the 20 infants in each group." |
| PMID | 9795454 |
| Year | 1998 |
| Species | Human |
| Journal | Acta Gastroenterol Belg |
| RefScore | 1 |
| Source | PArchNLP |
  ||  |  |


---

|  |  |
| --- | --- |
 Reference:24 || Sentence | The effects of compatibility, that is combination of Prunus persica Batsch (L.) and Carthamus tinctorus (L.), and different acidity of digestive site on the species, lipopily and bioavailability of coordinated complex of iron, manganese, and zinc in medical decoction were studied. |
| Year | 2004 |
| PMID | 14698191 |
| Species | Human |
| Journal | Bioorg Med Chem Lett |
| RefScore | 2 |
| Source | PArchNLP |
  ||  |  |


---

|  |  |
| --- | --- |
 Reference:25 || Sentence | BACKGROUND: Iron, zinc, and calcium can interact with each other in a way that inhibits their respective absorption. |
| Year | 2004 |
| PMID | 14749230 |
| Species | Human |
| Journal | Am J Clin Nutr |
| RefScore | 0 |
| Source | PArchNLP |
  ||  |  |


---

|  |  |
| --- | --- |
 Reference:26 || Sentence | The amyloid precursor protein binds copper and zinc in its extracellular domain, and the Abeta peptides also bind copper, zinc, and iron. |
| PMID | 15722349 |
| Year | 2005 |
| Species | Human |
|  | Rat |
| Journal | J Biol Chem |
| RefScore | 2 |
| Source | PArchNLP |
  ||  |  |


---

|  |  |
| --- | --- |
 Reference:27 || Sentence | Based on these results it is concluded that Fe, Co, Ni and Zn form stable octahedral complexes, whereas tetrahedral or square planar complexes are formed preferentially for other metals. |
| PMID | 15880669 |
| Year | 2005 |
| Journal | Rapid Commun Mass Spectrom |
| RefScore | 1 |
| Source | PArchNLP |
  ||  |  |


---

|  |  |
| --- | --- |
 Reference:28 || Sentence | Potential interactions between zinc and iron during absorption and its functional consequences on intestinal oxidative damage and antioxidant status were studied using the zinc-deficient rat as a model. |
| PMID | 16198227 |
| Year | 2005 |
| Species | Rat |
| Journal | Free Radic Biol Med |
| RefScore | 1 |
| Source | PArchNLP |
  ||  |  |


---

|  |  |
| --- | --- |
 Reference:29 || Sentence | Iron and zinc interacted to affect iron and zinc status, but not hemoglobin. |
| PMID | 16920862 |
| Year | 2006 |
| Species | Human |
| Journal | J Nutr |
| RefScore | 1 |
| Source | PArchNLP |
  |


---

|  |  |
| --- | --- |
